# Improved random forest classification model combined with C5.0 algorithm for vegetation feature analysis in non-agricultural environments

**DOI:** 10.1038/s41598-024-60066-x

**Published:** 2024-05-06

**Authors:** Tianyu Wang

**Affiliations:** College of Architecture, Nanjing Tech University, Nanjing City, 211800 China

**Keywords:** Random forest classification model, Vegetation feature analysis, Biodiversity, Multi-layer scale parameters, Plant sciences, Computer science, Information technology

## Abstract

In response to the challenges posed by the high computational complexity and suboptimal classification performance of traditional random forest algorithms when dealing with high-dimensional and noisy non-agricultural vegetation satellite data, this paper proposes an enhanced random forest algorithm based on the C5.0 algorithm. The paper focuses on the Liaohe Plain, selecting two distinct non-agricultural landscape patterns in Shenbei New District and Changtu County as research objects. High-resolution satellite data from GF-2 serves as the experimental dataset. This paper introduces an ensemble feature method based on the bagging concept to improve the original random forest classification model. This method enhances the likelihood of selecting features beneficial to classifying positive class samples, avoiding excessive removal of useful features from negative samples. This approach ensures feature importance and model diversity. The C5.0 algorithm is then employed for feature selection, and the enhanced vegetation index (EVI) is utilized for vegetation coverage estimation. Results indicate that employing a multi-scale parameter selection tool, combined with limited field-measured data, facilitates the identification and classification of plant species in forest landscapes. The C5.0 algorithm effectively selects classification features, minimizing information redundancy. The established object-oriented random forest classification model achieves an impressive accuracy of 94.02% on the aerial imagery for forest classification dataset, with EVI-based vegetation coverage estimation demonstrating high accuracy. In experiments on the same test set, the proposed algorithm attains an average accuracy of 90.20%, outperforming common model algorithms such as bidirectional encoder representation from transformer, FastText, and convolutional neural network, which achieve average accuracies ranging from 84.41 to 88.33% in identifying non-agricultural artificial habitat vegetation features. The proposed algorithm exhibits a competitive edge compared to other algorithms. These research findings contribute scientific evidence for protecting agricultural ecosystems and restoring agricultural ecosystem biodiversity.

## Introduction

The vegetation structure in non-agricultural environments plays a pivotal role in agricultural landscapes by providing habitat, food resources, and a platform for species interactions. Furthermore, it regulates ecosystem functions and supports biodiversity in agroecosystems^1,2^. Accurate identification and comprehensive understanding of vegetation characteristics and spatial distribution in non-agricultural environments are vital for preserving biodiversity in agroecosystems^3^.

Quantifying and describing the morphological, ecological, and physiological characteristics of plant species in non-agricultural environments is a fundamental step in studying vegetation characteristics^1,4^. Hinton et al.^5^ demonstrated the importance of non-agricultural vegetation in mitigating conflicts between humans and deer by studying the spatial utilization patterns of deer. They emphasized the need to protect and optimize the structure of non-agricultural vegetation to provide suitable habitats and food resources, reducing the dependence of deer on agricultural fields and minimizing conflicts with humans^5^. Suraci et al.^6^ employed a novel remote sensing estimation approach to quantify the impacts of agricultural management practices on bird habitats and migration. They revealed complex relationships between agriculture and key species, underscoring the influence of agricultural management on species habitats. Their study provided spatial recommendations for guiding agricultural management actions, contributing to the conservation and enhancement of biodiversity and ecosystem functionality in non-agricultural environments, and promoting harmonious coexistence between humans and nature^6^. Unmanned aerial vehicles (UAVs) have emerged as effective tools for estimating grassland biomass or vegetation cover, with diverse applications in studying vegetation characteristics^7^. Equipped with sensors such as multispectral and thermal infrared sensors, UAVs provide rich data for monitoring vegetation indices and other features. Advancements in UAV technology aim to improve spatial resolution, computing power, and image processing algorithms, enhancing data accuracy and precision^8^. Chen et al.^9^ investigated the use of aerial images acquired from UAV platforms for wetland vegetation and ground object classification. They determined optimal segmentation scale parameters by employing machine learning classifiers, such as random forest, support vector machine (SVM), K-nearest neighbors, and Bayesian methods. Their study explored variation patterns of vegetation characteristics and identified optimal spatial resolution images for wetland vegetation species and ground objects^9^. Buczyńska et al.^10^ demonstrated the utility of remote sensing images, when processed in a geographic information system, for studying the biophysical and biochemical parameters of plant communities. Remote sensing images provide spatial information on plant populations, enabling analysis of morphology, structure, distribution, and other features. Geographic information systems facilitate spatial and temporal analysis, data visualization, and integration of remote sensing data with other geographic datasets. This integration enables spatiotemporal correlation analysis, leading to a better understanding of the dynamic changes and ecological processes of plant communities^10^. However, limitations persist in the current research domain, notably the high computational complexity and suboptimal classification performance of traditional random forest algorithms when dealing with high-dimensional and noisy non-agricultural vegetation satellite data. Moreover, accurately identifying non-agricultural habitat vegetation features and acquiring spatial location information remains challenging. These constraints impede the scientific foundation for agricultural ecosystem protection and biodiversity restoration, necessitating urgent improvements and algorithm optimizations to enhance classification accuracy and precision.

This paper focuses on the Liaohe Plain, selecting Shenbei New District and Changtu County—representing distinct non-agricultural landscape patterns—as research areas. Leveraging high-resolution satellite data from GF-2, an ensemble feature method based on the bagging concept is proposed to enhance the original random forest classification model. This method increases the likelihood of selecting features conducive to classifying positive class samples and mitigates the issue of discarding useful features from negative samples excessively, thereby preserving feature importance and model diversity. Finally, the C5.0 algorithm is utilized for feature selection, and the enhanced vegetation index (EVI) is employed to estimate vegetation coverage. The innovation of this paper lies in the integration of the C5.0 algorithm and the enhanced random forest algorithm. The model’s classification accuracy is enhanced by incorporating ensemble feature methods, selecting classification features, and utilizing EVI for vegetation coverage estimation. This improvement facilitates the identification of non-agricultural artificial habitat vegetation features, providing a scientific basis for agricultural ecosystem protection and biodiversity restoration. This paper introduces the C5.0 algorithm and proposes an ensemble feature method based on the bagging concept, combined with high-resolution satellite data and multi-scale parameter selection tools. This approach aims to refine existing algorithms, accurately identify and classify vegetation species in agricultural landscapes, address current research limitations, enhance the accuracy of identifying non-agricultural artificial habitat vegetation features, and provide more reliable scientific support for sustainable agricultural ecosystem protection and effective biodiversity management.

## Literature review

With the rapid advancement of information technology, feature analysis algorithms have been continuously optimized in text analysis algorithms. Ozigis et al.^11^ conducted research on the fusion and classification of various vegetation indices and spectral wavelengths in different bands, utilizing random forest classifiers. The random forest-machine learning classifier demonstrates versatility in its application to various ecological environments and has the capability to generate accurate vegetation function type maps, thereby offering an effective approach for vegetation classification^12^. Dobrinić et al.^13^ employed a random forest variable selection method with reduced precision to identify the most relevant features for vegetation mapping, resulting in improved classification performance suitable for large-scale land cover classification. Meno et al.^14^ utilized machine learning algorithms such as random forest and C5.0 decision trees to successfully predict daily late blight spore levels, with the C5.0-optimized random forest model achieving higher accuracy. Guo et al.^15^ investigated the generation of regional landslide susceptibility maps using machine learning methods based on the C5.0 decision tree model and K-means clustering algorithm. Their results showed superior mapping outcomes compared to traditional models like SVMs and Bayesian networks^15^. Çelik^16^ conducted a comparison between the C4.5 and C5.0 algorithms and found that the classification model built using the C5.0 algorithm exhibited lower misclassification rates and higher accuracy. The use of satellite-derived normalized difference vegetation index (NDVI) and EVI enables the assessment of the direct impact of floods on vegetation cover, offering an effective method for studying vegetation coverage^17,18^. Additionally, Dai et al.^19^ demonstrated that the evaluation of the influence of crop residues on vegetation index and vegetation cover estimation could be achieved by comparing enhancement values and vertical values using a 2-m pixel model and a three-dimensional radiative transfer model.

In summary, machine learning algorithms, including random forest and C5.0 decision trees, have found extensive application in vegetation classification, land cover classification, and yield prediction. Additionally, the NDVI and EVI have emerged as popular indicators for assessing vegetation coverage. Nevertheless, the combined utilization and application of these methods in non-agricultural environments remain relatively limited, and there exist certain constraints on their use.

## Research theory and improved random forest model

### Habitat analysis

Habitat is defined as a distinct geographic area with a defined spatial extent and specific environmental conditions that offer essential resources and favorable conditions for the survival, reproduction, and life cycle completion of biological populations or individuals^20^.

### Non-agricultural habitat vegetation

**Figure 1:**
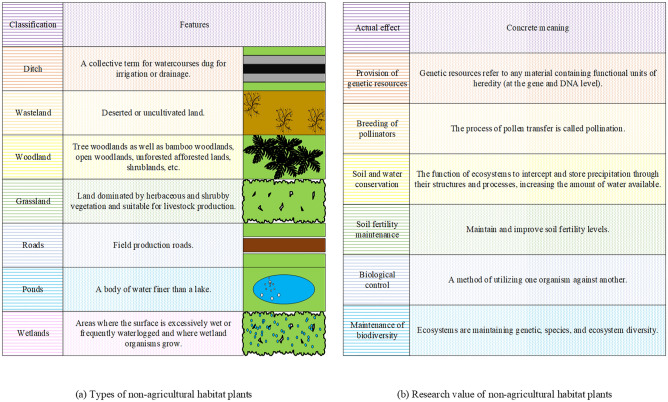
Categories and research value of non-agricultural environment vegetation.

Non-agricultural environment vegetation encompasses a wide range of plant communities found in non-agricultural settings. These vegetation types include diverse plant forms, such as flowers in urban parks, street trees along sidewalks, and trees and herbaceous plants in forests^21^. Figure 1 visually presents the different types of non-agricultural environment vegetation and highlights their research significance.

In non-agricultural environments, plant diversity (***S***_***W***_), richness (***F***), and dominance (***Y***) can be calculated using the following equations:

1 $${\varvec{S}}_{{\varvec{W}}} = - \mathop \sum \limits_{{{\varvec{M}} = 1}}^{{\varvec{N}}} {\varvec{Z}}_{{\varvec{M}}} \ln {\varvec{Z}}_{{\varvec{M}}}$$

2 $${\varvec{F}} = \frac{{{\varvec{N}} - 1}}{{\ln {\varvec{X}}}}$$

3 $${\varvec{Y}} = 1 - \sum {\varvec{Z}}_{{\varvec{M}}}^{2}$$

In these equations, ***N*** stands for the number of species in a sample plot, ***X*** signifies the total number of individuals of all species in the sample plot, and ***Z***_***M***_ represents the importance of species ***M*** within its population. These equations provide a quantitative assessment of plant diversity, richness, and dominance in non-agricultural environments.

The theoretical framework of habitats lays the groundwork for understanding the distribution, ecological functions, and impacts of non-agricultural habitat vegetation. Researchers can establish a comprehensive research background and theoretical framework by incorporating concepts of habitat and non-agricultural habitat vegetation. This enables targeted exploration of ecological characteristics, ecosystem functions, and classification issues pertaining to vegetation in non-agricultural habitats. Such a framework serves as the basis for addressing the challenges posed by high computational complexity and poor classification performance of traditional random forest algorithms in classifying non-agricultural habitat vegetation.

### High-resolution satellite-2 (GF-2) data processing workflow

The high-resolution satellite-2 (GF-2) is a domestically developed remote sensing satellite system in China that offers high-resolution and multispectral capabilities. It was designed and manufactured by the Fifth Academy of China Aerospace Science and Technology Corporation^22^. Detailed parameters of the GF-2 satellite can be found in Tables 1 and 2.

**Table 1:** Gaofen-2 satellite orbital parameters.

Project type	Parametric situation
Track type	Sun-synchronous return orbit
Track height	631 km
Inclination size	97.9080°
Drop node local time	10:30 am
Side swing capacity	± 35°

**Table 2:** GF-2 satellite sensor parameters.

Parameter	Camera situation
Spectral range	
Full color	0.45–0.9 μm
Multi-spectrum	0.45–0.52 μm
	0.52–0.59 μm
	0.63–0.69 μm
	0.77–0.89 μm
Spatial resolution	
Full color	0.8 m
Multi-spectrum	3.2 m
Width	45 km
Revisit period with side swing	5 days
Revisit period without side swing	69 days

**Figure 2:**
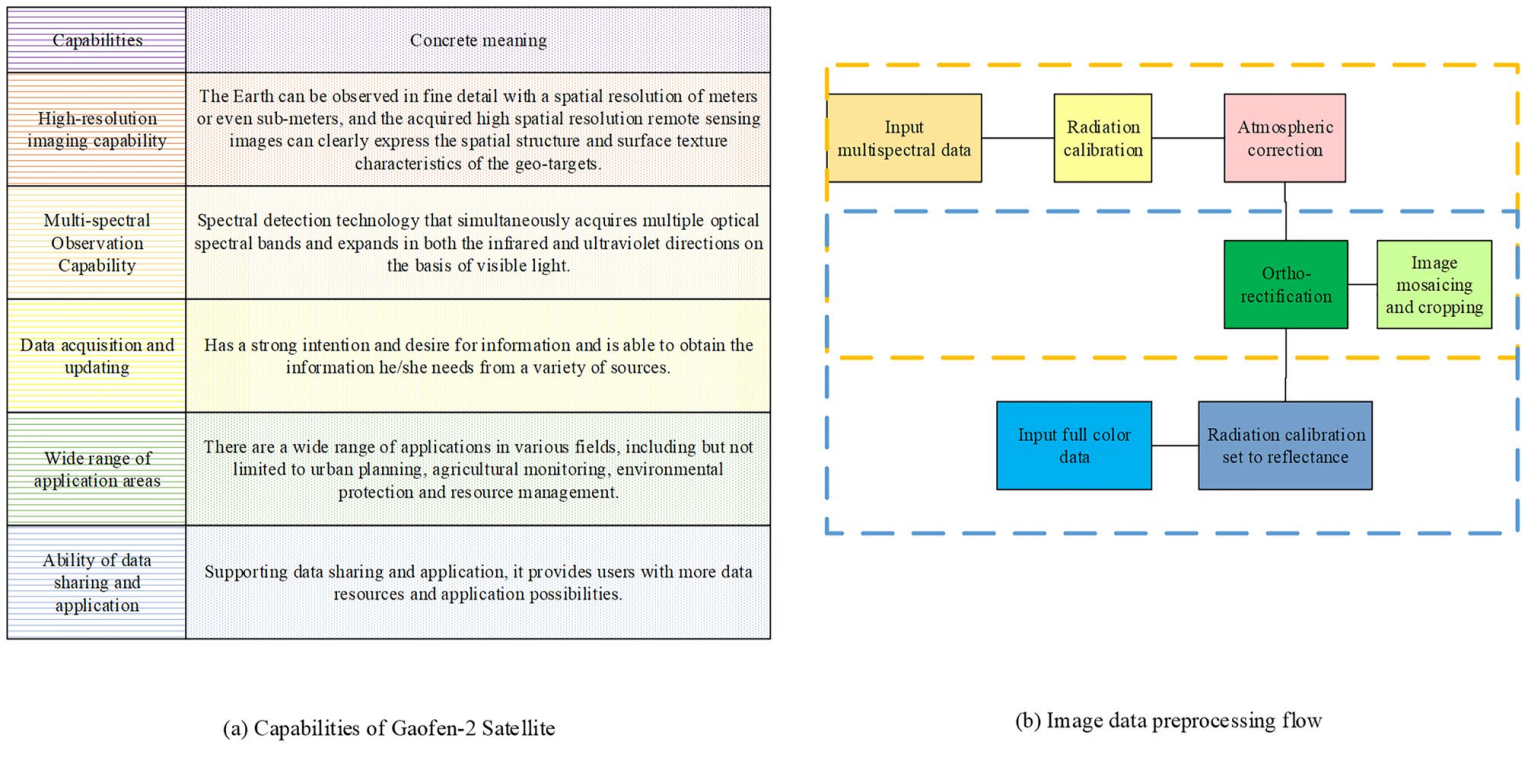
Capability of GF-2 satellite and image data preprocessing process.

The GF-2 satellite has significantly contributed to diverse fields, including land resource surveys and environmental monitoring, by providing high-resolution multispectral image data. This has been made possible through the implementation of an efficient image data preprocessing workflow tailored specifically for the GF-2 satellite. The extensive capabilities of the GF-2 satellite, along with its accompanying image data preprocessing workflow, are clearly illustrated in Fig. 2.

Figure 2 showcases the remarkable capabilities of the GF-2 satellite, including high-resolution imaging, multispectral observation, data acquisition and updates, wide application domains, as well as data sharing and utilization.

### C5.0 algorithm and computational process

**Figure 3:**
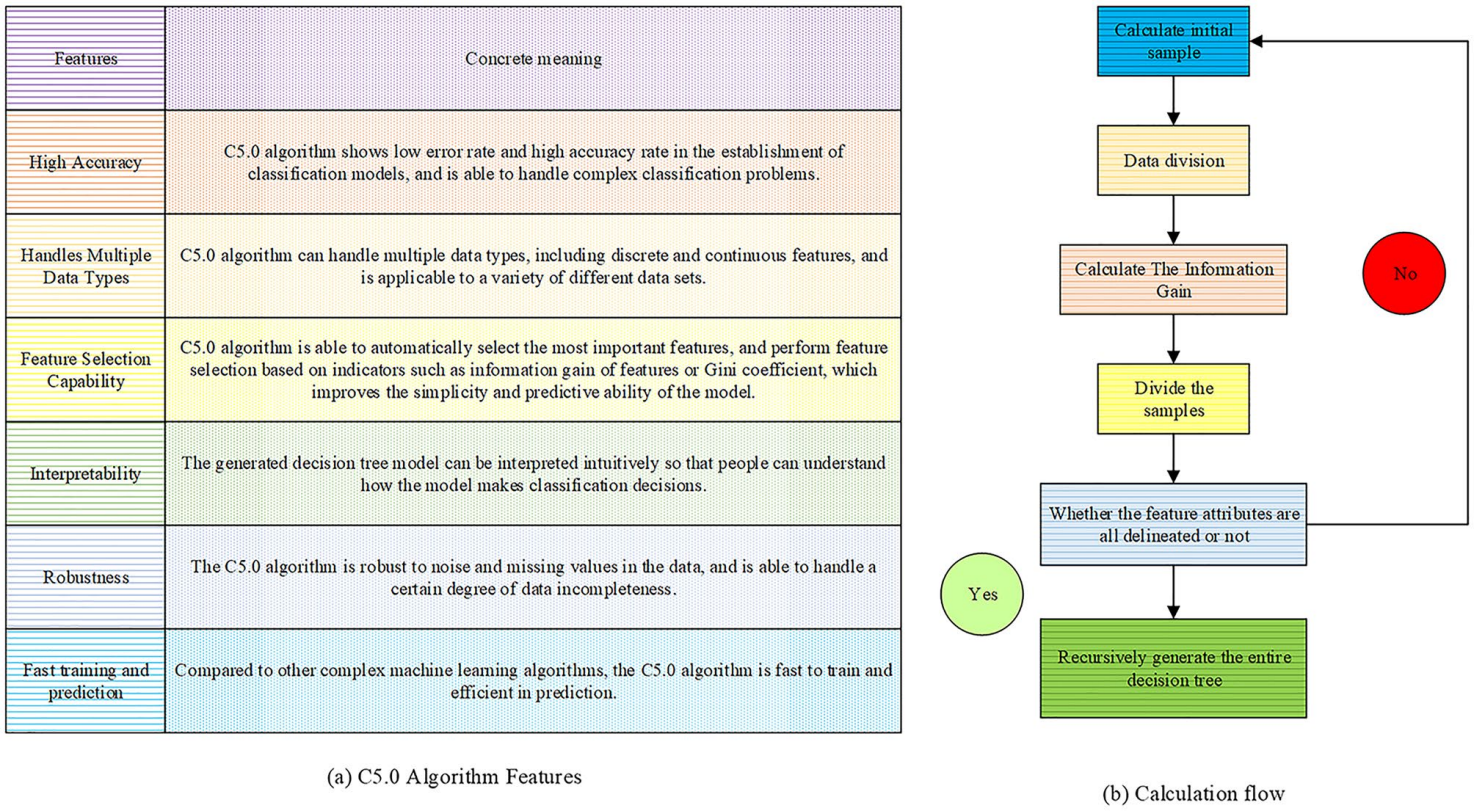
C5.0 algorithm features and calculation flow.

The C5.0 algorithm is a decision tree algorithm that utilizes the information gain ratio criterion for effective analysis. It is particularly suitable for handling high-dimensional data and large-scale datasets. Through the process of feature selection and determination of splitting points, the C5.0 algorithm efficiently extracts valuable information from complex data structures^23^. The key characteristics and computational process of the C5.0 algorithm are visually represented in Fig. 3.

As illustrated in Fig. 3, the C5.0 algorithm exhibits distinct characteristics, including feature selection, determination of splitting points, and recursive processing. It excels in constructing decision tree models that are both accurate and interpretable, enhancing model generalization through the implementation of pruning operations. The computational process primarily entails data initialization, feature selection, data splitting, and recursive processing.

### Estimation of EVI and calculation of vegetation cover

**Figure 4:**
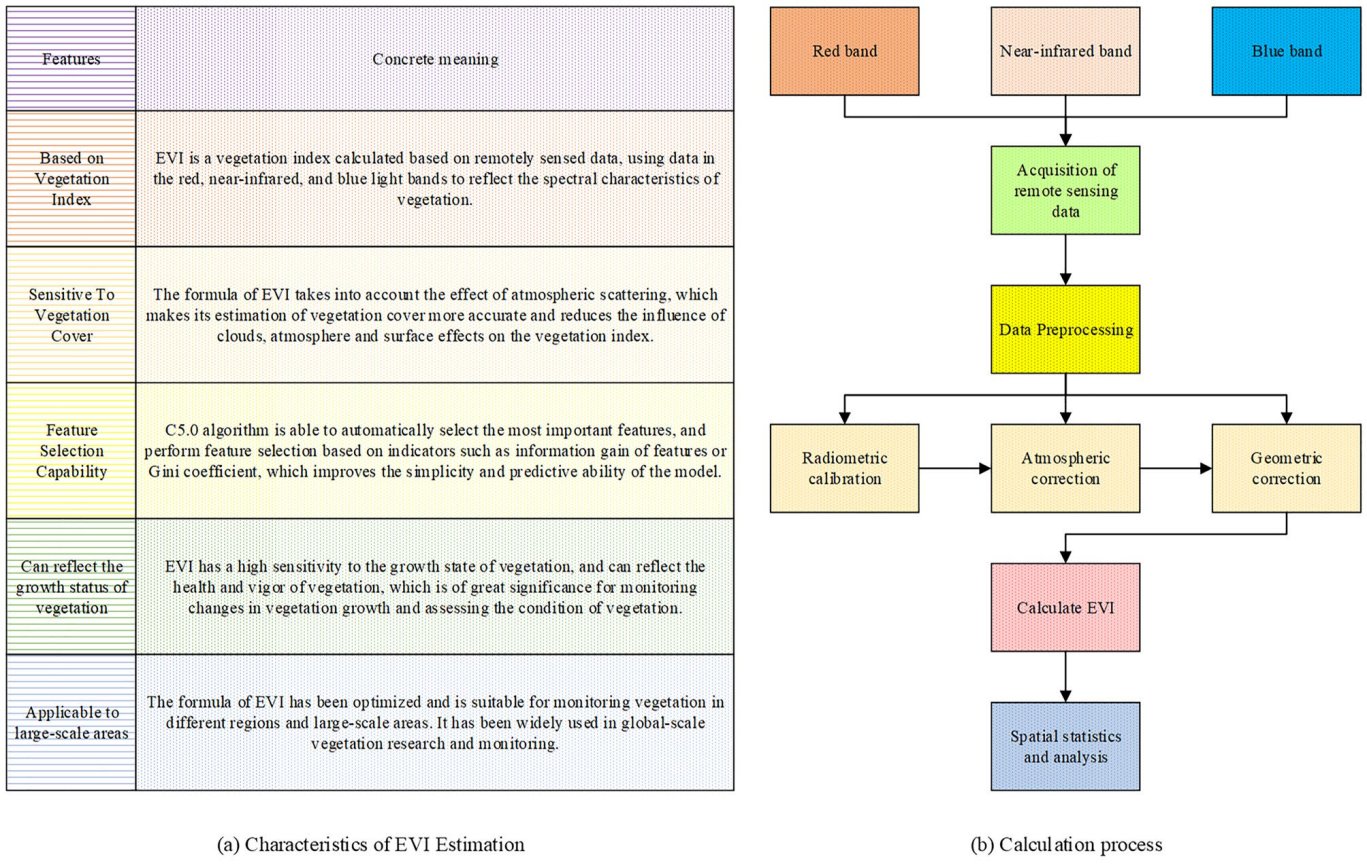
Characteristics and computational process of EVI estimation.

The estimation of vegetation cover is accomplished using the EVI, which utilizes remote sensing data from the visible and near-infrared bands^24^. EVI serves as an effective index for assessing vegetation cover and is characterized by specific computational processes, as depicted in Fig. 4.

As depicted in Fig. 4, EVI estimation possesses distinct characteristics, including its reliance on vegetation indices, sensitivity to vegetation cover, ability to reflect vegetation growth status, and applicability to large-scale areas. The computational process of EVI estimation encompasses several stages, namely obtaining remote sensing data, performing data preprocessing, calculating EVI values, conducting spatial statistics and analysis, and interpreting and applying the obtained results.

Let ***R***_***r***_ represent the reflectance of near-infrared light in the remote sensing image, ***r*** denotes the reflectance of red light in the remote sensing image, and ***b*** indicates the reflectance of blue light in the remote sensing image. ***O*** signifies the gain factor used to correct spectral response, ***V***_**1**_ and ***V***_**2**_ serve as adjustment parameters used to correct atmospheric scattering and soil background effects, and ***D*** stands for the adjustment parameter for correcting image background brightness. The EVI is defined as Eq. (4).

4 $${\varvec{E}} = \frac{{{\varvec{O}}\left( {{\varvec{R}}_{{\varvec{r}}} - {\varvec{r}}} \right)}}{{{\varvec{R}}_{{\varvec{r}}} + {\varvec{V}}_{1} {\varvec{r}} - {\varvec{V}}_{2} {\varvec{b}} + {\varvec{D}}}}$$

Vegetation cover refers to the extent or proportion of a particular region or surface that is occupied by plants. It provides information about the density and growth status of vegetation in that area^25^. The estimation of vegetation cover is commonly performed using methods such as the NDVI and EVI algorithms. These indices enable the quantification and assessment of vegetation abundance and health.

The determination coefficient ***K***^**2**^ and root mean square error ***W*** can be used to evaluate the accuracy of vegetation cover estimation. The equations for calculating ***K***^**2**^ and ***W*** are as follows, where ***q*** represents the total number of samples, ${\acute{\varvec{j}}}_{{\varvec{p}}}$ denotes the vegetation cover value for the ***p***th sample, ${\acute{\varvec{J}}}$ represents the modeled estimation of the vegetation cover value for the ***p***th sample, and $\overline{\varvec{j}}$ denotes the average vegetation cover value:

5 $${\varvec{K}}^{2} = \frac{{\mathop \sum \nolimits_{{{\varvec{p}} - 1}}^{{\varvec{q}}} \left( {{\acute{\varvec{J}}} - \overline{\varvec{j}}} \right)^{2} }}{{\mathop \sum \nolimits_{{{\varvec{p}} - 1}}^{{\varvec{q}}} \left( {{\acute{\varvec{j}}}_{{\varvec{p}}} - \overline{\varvec{j}}} \right)^{2} }}$$

6 $${\varvec{W}} = \sqrt {\frac{{\mathop \sum \nolimits_{{{\varvec{p}} - 1}}^{{\varvec{q}}} \left( {{\varvec{J}}_{{\varvec{p}}} - {\acute{\varvec{j}}}_{{\varvec{p}}} } \right)^{2} }}{{\varvec{q}}}}$$

### Random forest classification model under text classification

**Figure 5:**
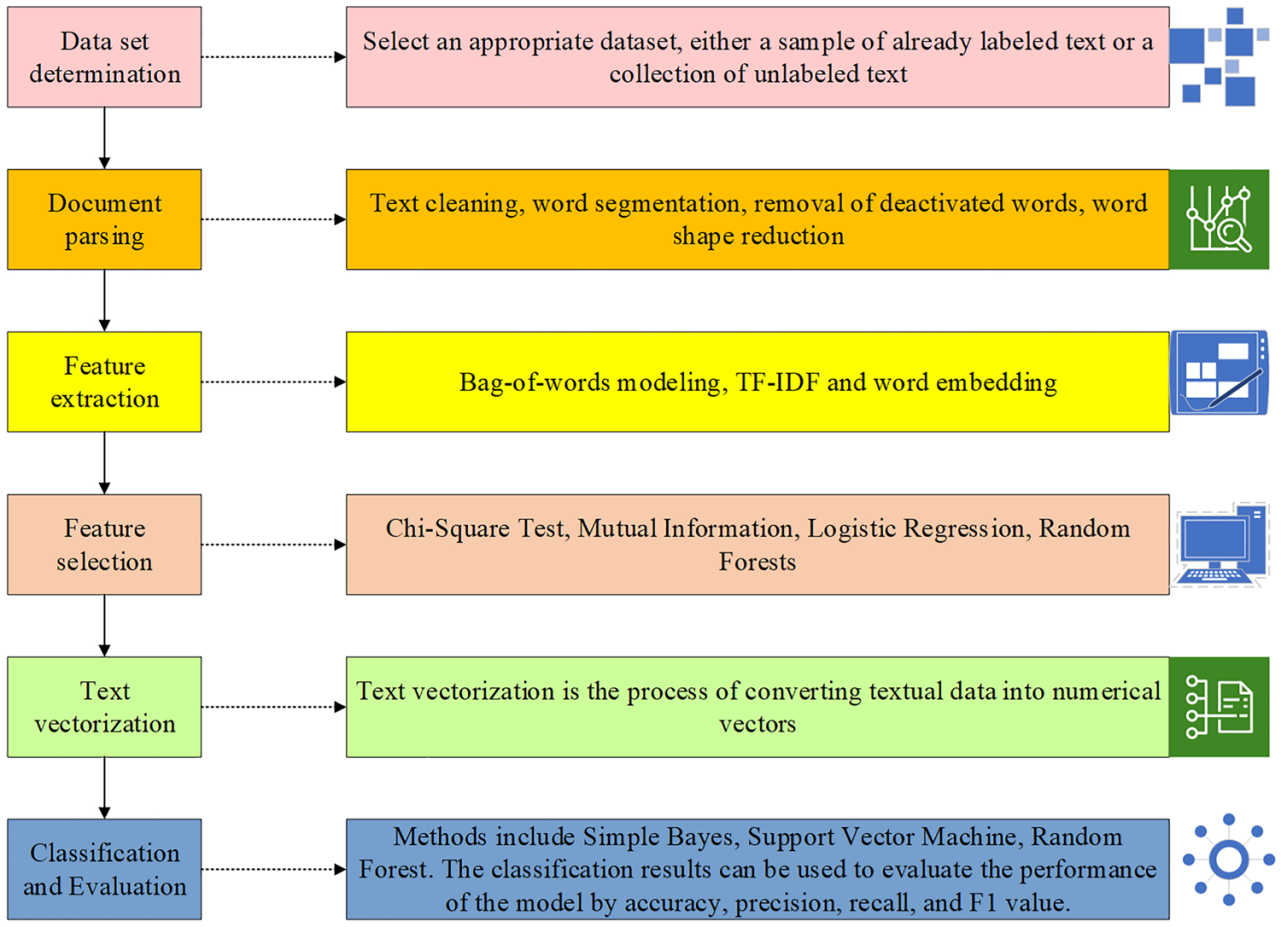
Text classification process.

Text classification is an automated process that aims to categorize textual data into predefined classes or labels. It encompasses several steps, including preprocessing the raw text, feature extraction, and training or predicting using machine learning or deep learning models^26^. The workflow for text classification is visualized in Fig. 5, demonstrating the sequence of tasks involved in the process.

As depicted in Fig. 5, the text classification process comprises several essential steps, including dataset selection, document parsing, feature extraction, feature selection, text vectorization, classification, and evaluation. Each of these steps plays a crucial role in achieving accurate and reliable text classification results.

**Figure 6:**
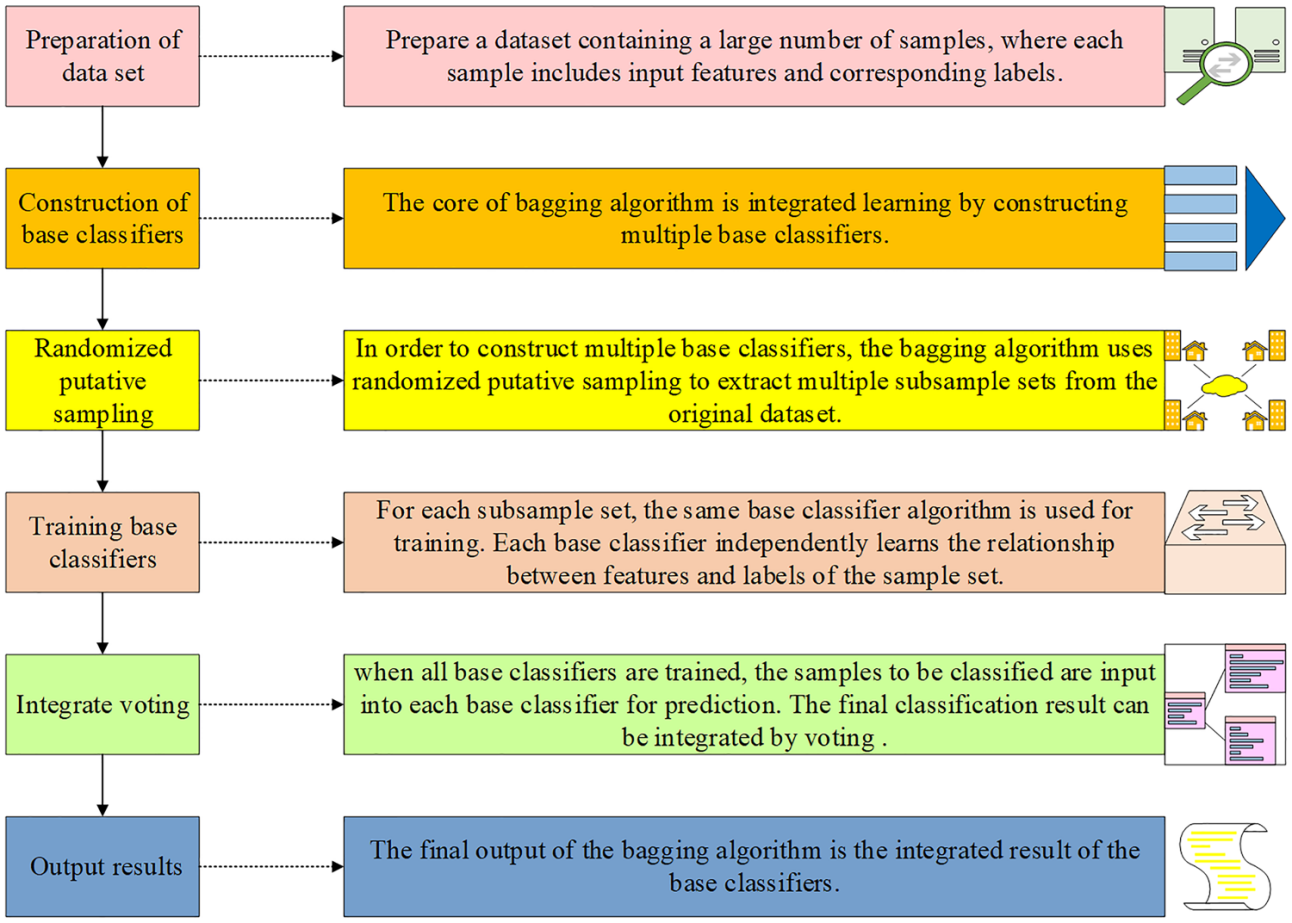
Calculation process of the bagging algorithm.

The bagging algorithm, illustrated in Fig. 6, is an ensemble method that effectively reduces model variance, improves generalization, and enhances prediction accuracy. It achieves this by employing bootstrap sampling and aggregation techniques. The bagging algorithm is widely used in various machine learning tasks and has been proven to provide stable and robust predictions through the combination of independent ensemble models^27^. Its computational process involves the creation of multiple subsets of the training dataset, training individual models on each subset, and aggregating their predictions to obtain the final classification outcome.

As depicted in Fig. 6, the bagging algorithm enhances the accuracy and stability of the model by combining multiple independent learners through bootstrap sampling, model training, and ensemble prediction.

**Figure 7:**
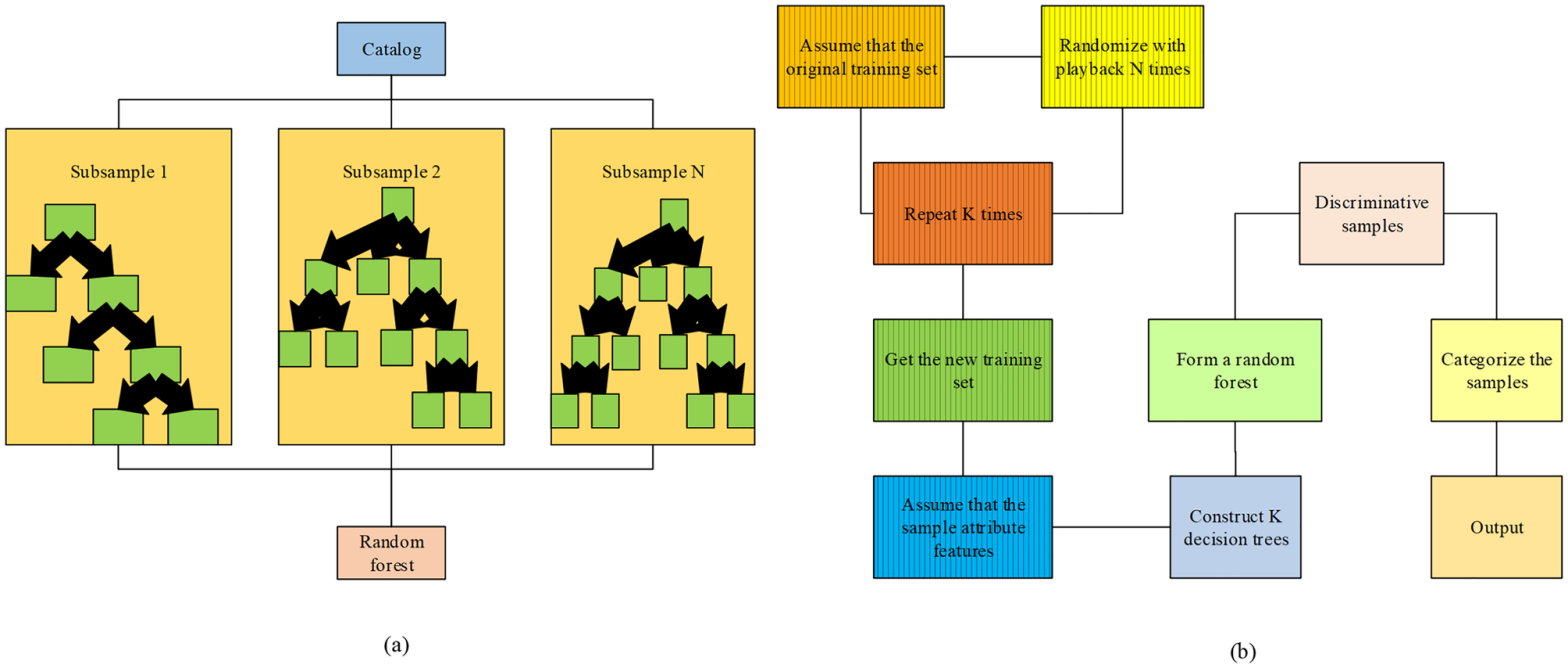
Structure and algorithm flow of the Random Forest model.

Random Forest is a robust machine learning algorithm widely employed for text classification tasks^28,29^. It exhibits notable performance in handling high-dimensional data and provides effective feature selection and prediction capabilities. Figure 7 illustrates the structure and algorithmic process of the random forest model.

Figure 7 depicts the Random Forest model comprising multiple decision trees. Each decision tree is trained using bootstrap sampling and random feature selection. The final classification or regression is conducted by aggregating the prediction results of individual trees, either through voting or averaging. This ensemble approach aims to enhance the accuracy and generalization capability of the model.

Let ***C*** represent the total number of pixels actually classified as class ***r***, ***c*** stands for the total number of pixels, ***β***_***vv***_ denotes the number of pixels correctly classified as class ***v***, ***β***_***rv***_ signifies the number of pixels misclassified as class ***r*** but actually belong to class ***v***, ***β***_***rr***_ represent the number of pixels correctly classified as class ***r***, and ***β***_***vr***_ represent the number of pixels classified as class r but actually belong to class ***v***. The formulas for overall accuracy ***Q***_***J***_, map accuracy ***Z***_***T***_, and user's accuracy ***Y***_***H***_ are as follows:

7 $${\varvec{QJ}} = \frac{{\mathop \sum \nolimits_{{{\varvec{v}} = 1}}^{{\varvec{c}}} {\varvec{\beta}}_{{{\varvec{vv}}}} }}{{\mathop \sum \nolimits_{{{\varvec{r}},{\varvec{v}} = 1}}^{{\varvec{c}}} {\varvec{\beta}}_{{{\varvec{rv}}}} }} - \frac{{\mathop \sum \nolimits_{{{\varvec{v}} = 1}}^{{\varvec{c}}} {\varvec{\beta}}_{{{\varvec{vv}}}} }}{{\varvec{c}}}$$

8 $${\varvec{ZT}} = \frac{{{\varvec{\beta}}_{{{\varvec{rr}}}} }}{{\mathop \sum \nolimits_{{{\varvec{r}} = 1}}^{{\varvec{C}}} {\varvec{\beta}}_{{{\varvec{vr}}}} }}$$

9 $${\varvec{YH}} = \frac{{{\varvec{\beta}}_{{{\varvec{rr}}}} }}{{\mathop \sum \nolimits_{{{\varvec{r}} = 1}}^{{\varvec{C}}} {\varvec{\beta}}_{{{\varvec{rv}}}} }}$$

Let ***T*** represent the total number of pixels used for accuracy evaluation, α represent the total number of classes, ***X***_***γγ***_ represent the number of correctly classified pixels, ***X***_***γg***_ represents the total number of pixels in the ***γ***th row of the confusion matrix, and ***X***_***gγ***_ represents the total number of pixels in the ***γ***th column of the confusion matrix. The kappa coefficient can be described as Eq. (10).

10 $${\varvec{KAP}} = \frac{{{\varvec{T}}\mathop \sum \nolimits_{{{\varvec{\gamma}} - 1}}^{{\varvec{\alpha}}} {\varvec{X}}_{{\varvec{\gamma \gamma }}} - \mathop \sum \nolimits_{{{\varvec{\gamma}} - 1}}^{{\varvec{\alpha}}} \left( {{\varvec{X}}_{{\varvec{\gamma g}}} {\varvec{X}}_{{\varvec{g\gamma }}} } \right)}}{{{\varvec{T}}^{2} - \mathop \sum \nolimits_{{{\varvec{\gamma}} - 1}}^{{\varvec{\alpha}}} \left( {{\varvec{X}}_{{\varvec{\gamma g}}} {\varvec{X}}_{{\varvec{g\gamma }}} } \right)}}$$

This paper enhances the random forest classifier by integrating steps from the C5.0 algorithm to boost its performance. The incorporation of the C5.0 algorithm leads to improvements in the random forest classifier's performance. Firstly, the implementation involves computing the entropy of initial samples to gauge information uncertainty. Subsequently, data partitioning is based on each feature, with the best splitting feature selected through information gain calculation. Following this, samples with the highest information gain ratio are chosen for partitioning, forming child nodes, and recursively generating the entire decision tree until all feature attributes are partitioned. These steps enhance the accuracy of the random forest classifier as the C5.0 algorithm efficiently selects splitting features, resulting in a more discriminative decision tree structure. By amalgamating the C5.0 algorithm with random forest, the improved algorithm better accommodates the high-dimensional and high-noise characteristics of non-agricultural habitat satellite data, thereby yielding more precise classification outcomes.

### Experimental data design

The improved random forest classification model based on the C5.0 algorithm established in this paper utilizes several databases, including the GF-2, Landsat, and Aerial Imagery Forest Classification (AIFC) datasets. The GF-2 database comprises high-resolution remote sensing image data, remote sensing products, and remote sensing application services from the Chinese High-Resolution Earth Observation System’s GF-2 satellite. The Landsat database contains remote sensing image data acquired through the United States Landsat program, which utilizes multispectral remote sensing technology to capture surface images and provides data for multiple spectral bands, widely applied in fields such as land use, vegetation monitoring, and water resources management. The AIFC dataset, available at (https://www.gisrsdata.com), is specifically designed for forest classification research, comprising high-resolution aerial imagery data tailored for forest areas, which can be used to train and evaluate the performance of forest classification algorithms and models (Supplementary information).

This paper utilizes multi-temporal remote sensing data from the GF-2 (Gaofen-2) and Landsat-8 satellites. GF-2 satellite data comprised panchromatic and multispectral bands, spanning 0.45–0.90 µm for the panchromatic band and including blue, green, red, and near-infrared bands for multispectral data. Landsat-8 satellite data encompasses multispectral bands, covering blue, green, red, and near-infrared bands. Observation times were GF-2 (2018-06-03) and Landsat-8 (2018-05-24). Initially, radiometric calibration using the Generic Calibrator tool in ENVI 5.3 software ensures data accuracy for both panchromatic and multispectral images. Subsequently, atmospheric correction on multispectral data is conducted using the FLASH tool to mitigate atmospheric and lighting effects on land feature reflectance. Orthorectification via the RPC Orthorectification Workflow in ENVI software eliminates geometric distortions, yielding accurate orthorectified images. Finally, multispectral image fusion with the panchromatic image using the GS method produced high-resolution multispectral images, serving as reliable foundational data for subsequent land cover classification and change detection. Feature extraction on segmented objects covers four main aspects: spectral, geometric, texture, and remote sensing indices, totaling 85 features. Spectral features, reflecting object spectral information, include grayscale mean, standard deviation, brightness, and maximum difference calculations. Geometric features, derived from covariance matrix statistics, describe an object’s geometric shape and size, comprising area, perimeter, length–width ratio, density, and rectangular fit. Texture features, calculated using a gray-level co-occurrence matrix and gray-level difference vector, capture object texture information, such as homogeneity, variance, heterogeneity, angular second moment, and entropy. Remote sensing indices, including NDVI, EVI, Atmospherically Resistant Vegetation Index (ARVI), Water Index, and Building Area Index, aided in land feature extraction.

This paper employs high-resolution satellite imagery data alongside an enhanced random forest classification model based on the C5.0 algorithm. To adapt text classification algorithms to image data, a preprocessing step is essential, transforming images into feature vectors suitable for algorithmic processing. This process entails extracting features like spectral information and texture features, alongside data preprocessing and labeling. Subsequently, appropriate text classification algorithms, such as SVMs and naive Bayes, are chosen for model training, leveraging enhanced feature selection methods and feature-based enhanced vegetation indices for optimization. Following model training, thorough evaluation and validation refine the classification model, which is then applied to unknown image data for prediction. This holistic approach effectively applies text classification algorithms to image data, enabling precise classification and identification of complex image data. The paper concentrates on forest classification research across two categories: forest and grassland. It employs 932 grassland samples and 45 forest samples for the training set, and 1031 grassland samples and 23 forest samples for the validation set, meticulously annotated and labeled to ensure the accuracy and reliability of the models presented in this paper.

## Result analysis of random forest classification model based on C5.0 algorithm

### Analysis of the accuracy results of the improved Random Forest classification model

**Figure 8:**
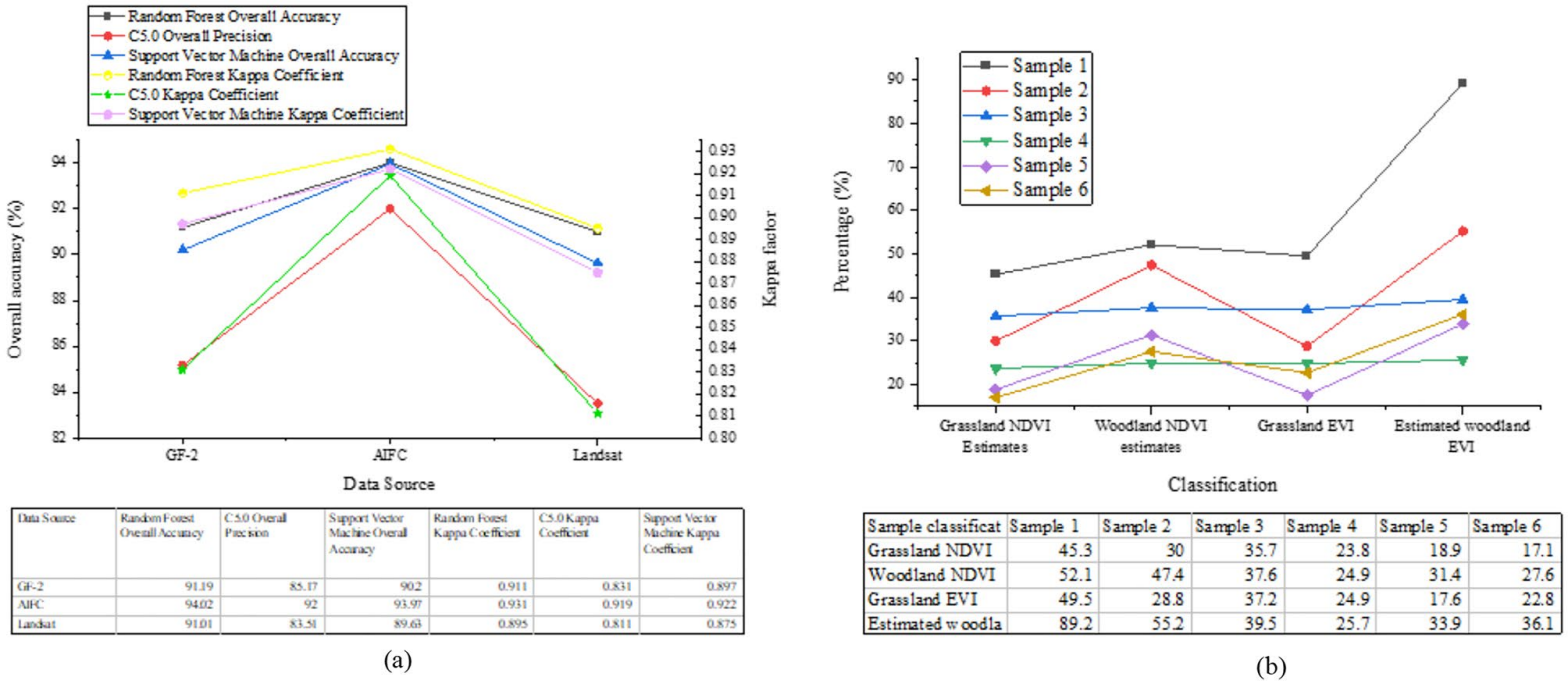
Comparison of model accuracy and vegetation coverage estimation results.

Figure 8 presents a comparison of the accuracy of the improved random forest classification model and the estimation results of vegetation coverage.

As depicted in Fig. 8, the improved Random Forest classification model achieves high accuracy on different datasets. On the GF-2 dataset, the Random Forest model exhibited an overall accuracy of 91.19% with a Kappa coefficient of 0.911. The C5.0 model achieves an overall accuracy of 85.17% with a Kappa coefficient of 0.831, while the SVM model achieves an overall accuracy of 90.2% with a Kappa coefficient of 0.897. On the AIFC dataset, the Random Forest model achieves an overall accuracy of 94.02% with a Kappa coefficient of 0.931, while the C5.0 model achieves an overall accuracy of 92% with a Kappa coefficient of 0.919. The SVM model achieves an overall accuracy of 93.97% with a Kappa coefficient of 0.922. On the Landsat dataset, the Random Forest model achieves an overall accuracy of 91.01% with a Kappa coefficient of 0.895. The C5.0 model achieves an overall accuracy of 83.51% with a Kappa coefficient of 0.811, while the SVM model achieves an overall accuracy of 89.63% with a Kappa coefficient of 0.875. The comparison of vegetation coverage estimation results indicates that different vegetation types have a significant impact on NDVI and EVI estimation values. Forested areas generally exhibit higher NDVI and EVI values compared to grassland, indicating a higher vegetation coverage and growth vitality in forested regions. Among the grassland samples, Sample 1 demonstrates the highest NDVI and EVI estimation values, measuring 45.3% and 49.5%, respectively, while Sample 5 exhibits the lowest values at 18.9% and 17.6%, respectively. Among the forest samples, Sample 1 has the highest NDVI and EVI estimation values at 52.1% and 89.2%, respectively, while Sample 6 has the lowest values at 27.6% and 36.1%, respectively. Notably, EVI estimation values generally outperform NDVI in reflecting the vegetation condition in forested areas, as they tend to be higher in such regions. In summary, the findings of this paper underscore the notable advantages of the enhanced random forest classification model in processing high-resolution satellite data. Across diverse datasets, the model exhibits high accuracy and Kappa coefficients, showcasing its proficiency in accurately categorizing various land cover types. Comparative analyses of NDVI and EVI estimates across different vegetation types unveil disparities in vegetation coverage and vitality, offering valuable insights into land surface vegetation distribution and ecosystem conditions. Moreover, the research outcomes emphasize the reliability and robustness of the enhanced random forest classification model in vegetation classification and coverage estimation, thereby furnishing substantial support for leveraging remote sensing data in ecological environment monitoring and resource management endeavors.

### Analysis of the evaluation accuracy results of the improved random forest classification model

**Figure 9:**
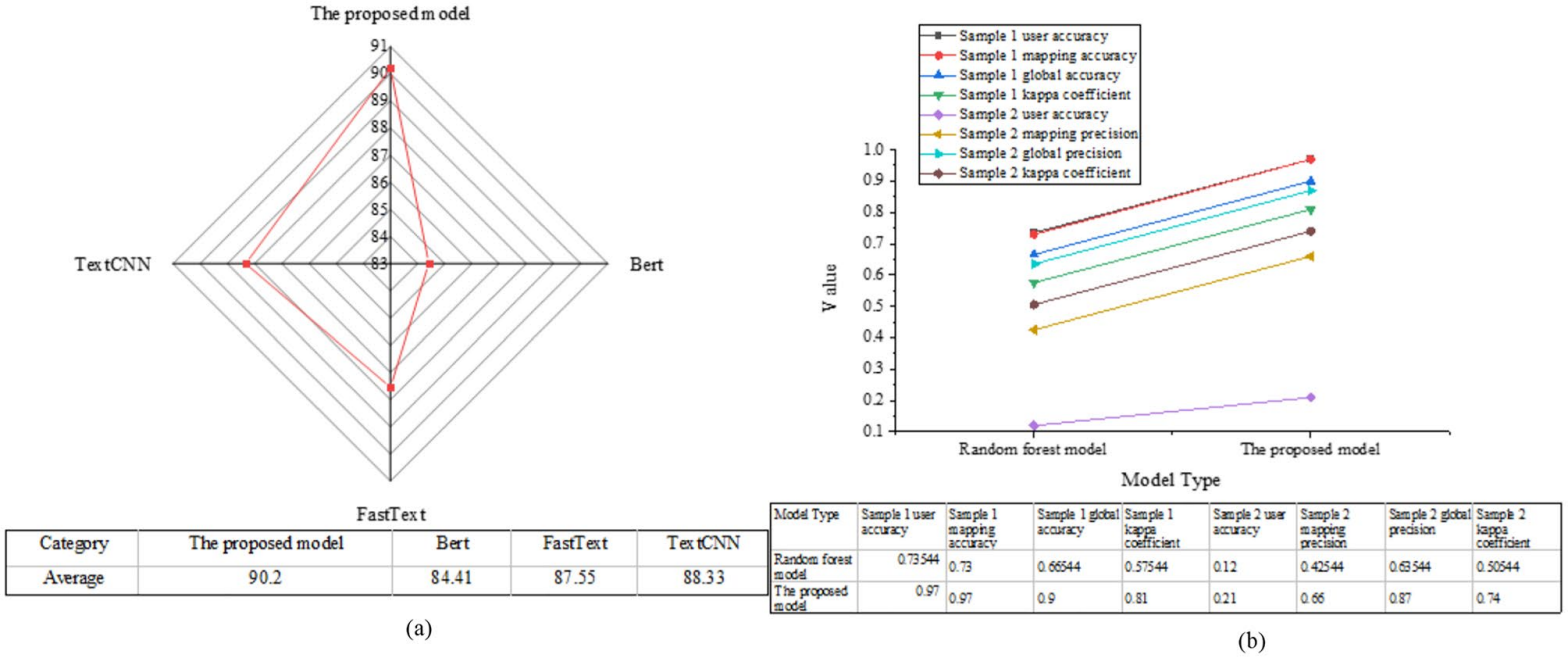
Comparison results of average accuracy and landscape classification accuracy of different models.

Figure 9 illustrates the comparison of average accuracy among different models and the landscape classification accuracy.

As depicted in Fig. 9, the comparison results of average accuracy among common classification models show that Bert, FastText, and TextCNN achieve average accuracies of 84.41%, 87.55%, and 88.33%, respectively. In contrast, the improved model algorithm attains an average accuracy of 90.20%, significantly outperforming these common models in recognizing features of non-agricultural artificial habitat vegetation. This underscores its superior classification accuracy and performance in non-agricultural habitat vegetation classification. Analyzing the landscape classification accuracy of the improved random forest model reveals notable enhancements. In Sample 1, the unimproved random forest model yields user accuracy, mapping accuracy, and overall accuracy of 0.74, 0.73, and 0.67, respectively, with a Kappa coefficient of 0.58, indicating subpar accuracy and performance. In contrast, the improved model achieves substantial improvements, with user accuracy, mapping accuracy, and overall accuracy reaching 0.97, 0.97, and 0.90, respectively. The Kappa coefficient rises to 0.81, signifying higher classification accuracy and result reliability. In Sample 2, the unimproved random forest model exhibits user accuracy, mapping accuracy, and overall accuracy of 0.12, 0.43, and 0.64, respectively, with a Kappa coefficient of 0.51, indicating inadequate overall performance. Conversely, the improved model demonstrates enhanced accuracy metrics, with user accuracy, mapping accuracy, overall accuracy, and Kappa coefficient reaching 0.21, 0.66, 0.87, and 0.74, respectively. These results affirm its superior overall accuracy and improved classification accuracy and result reliability. In general, the evaluation and comparison of the enhanced random forest classification model in non-agricultural artificial habitat vegetation classification tasks yield the following conclusions: The enhanced model exhibits remarkable accuracy and reliability in discerning non-agricultural artificial habitat vegetation characteristics. Compared to conventional classification models, it attains higher average accuracy, signifying superior classification performance. Furthermore, through comprehensive landscape classification accuracy analysis, substantial enhancements are observed across various samples, further affirming its efficacy in practical scenarios. In summary, this enhanced random forest classification model holds considerable practical value and promising application prospects, particularly in ecological environment monitoring, resource management, and land use planning.

## Discussion

In the realm of non-agricultural habitat vegetation research, this paper delves deeply into the classification of vegetation satellite data within non-agricultural environments. Focusing on the Liaohe Plain and two distinct non-agricultural landscapes, Shenyang North New District and Changtu County, high-resolution satellite data serve as the experimental dataset. The prevalent challenges of high dimensionality and significant noise are acknowledged in the field. However, through refining the random forest classification model and integrating the C5.0 algorithm and EVI estimation, this paper aims to optimize the feature analysis model, enhancing the accuracy and generalization ability of the classification model for non-agricultural habitat vegetation. Notably, the adoption of an ensemble feature method based on the bagging approach increases the likelihood of selecting features conducive to classifying positive samples while mitigating the risk of discarding useful features from negative samples. This ensures the significance of features and promotes model diversity, offering a novel approach to address issues like information redundancy and high computational complexity in satellite data classification for non-agricultural habitat vegetation. Additionally, leveraging the C5.0 algorithm alongside EVI estimation provides a more scientific foundation for selecting classification features. Overall, this paper innovates in methodology and demonstrates superior accuracy and competitiveness through experimentation in classifying non-agricultural habitat vegetation. By enhancing the capability to identify and classify such vegetation, it furnishes a more reliable scientific underpinning for ecosystem protection and biodiversity restoration in farmland ecosystems. Future research avenues could explore the applicability of this method in diverse regions and datasets to affirm its universality and stability. Research on non-agricultural habitat vegetation serves multiple purposes, including comprehending urban ecosystems, preserving natural environments, assessing vegetation health, and providing scientific grounding for urban planning, ecological conservation, and sustainable development.

## Conclusion

In recent years, the impact of non-agricultural habitat vegetation on ecological diversity and balance has grown in significance. However, challenges persist in satellite data classification of such vegetation, prompting the need for research to optimize models for feature analysis, enhancing classification accuracy. This paper selects the Liaohe Plain as the research area, with Shenyang North New District and Changtu County as focal points, utilizing high-resolution satellite data as the experimental dataset. The original random forest model is refined to improve classification by introducing an ensemble feature method based on the bagging approach. This method enhances the selection of features conducive to classifying positive samples while preserving useful features from negative samples, ensuring feature importance and model diversity. Additionally, the C5.0 algorithm is employed for feature selection, and EVI is utilized to estimate vegetation coverage. The results demonstrate the high classification performance of the random forest model in non-agricultural habitat vegetation satellite data classification. Achieving an overall accuracy of 94.02% and a Kappa coefficient of 0.931 on the AIFC dataset, the random forest model outperforms the C5.0 model and support vector machine model in terms of classification accuracy and reliability. Moreover, EVI-based vegetation coverage estimation yields highly accurate results. With an average accuracy of 90.20%, the improved algorithm surpasses common model algorithms like Bert, FastText, and TextCNN, which had average accuracies ranging from 84.41 to 88.33%. This underscores the enhanced accuracy of the improved model algorithm, rendering it more adept at identifying features of non-agricultural habitat vegetation. The enhanced model facilitates precise identification and mapping of target categories, offering valuable insights for decision-making and resource management in relevant fields. It also provides guidance for further refinement and application of classification algorithms, contributing to advancements in satellite data analysis and ecosystem management.

One limitation of this paper pertains to the data used. The experiments were conducted solely on specific regions and agricultural landscape data from the Liaohe Plain, which may introduce biases and restrict the representation of a broader range of non-agricultural habitat vegetation. Furthermore, the parameter settings employed here may not be universally applicable to other datasets, necessitating further investigation into parameter tuning and generalizability studies. To address this limitation, future research should aim to expand the dataset by incorporating a wider range of non-agricultural habitat vegetation types from diverse regions. This strategy would facilitate the validation of the improved algorithm's robustness and applicability. Additionally, optimizing the parameter settings of the improved algorithm should be considered to enhance model performance and generalizability, enabling its suitability for various non-agricultural habitat vegetation classification tasks. Lastly, exploring the integration of additional text classification algorithms or incorporating deep learning methods could be explored to further enhance the accuracy and applicability of non-agricultural habitat vegetation classification.

### Supplementary Information

Supplementary Information.

## Data Availability

The data used to support the findings of this study are included within the article.
